# Perceptual learning of second order cues for layer decomposition

**DOI:** 10.1016/j.visres.2012.11.005

**Published:** 2013-01-25

**Authors:** Dicle N. Dövencioğlu, Andrew E. Welchman, Andrew J. Schofield

**Affiliations:** School of Psychology, University of Birmingham, Edgbaston, Birmingham B15 2TT, UK

**Keywords:** Second-order, Layer-decomposition, Perceptual-learning

## Abstract

Luminance variations are ambiguous: they can signal changes in surface reflectance or changes in illumination. Layer decomposition—the process of distinguishing between reflectance and illumination changes—is supported by a range of secondary cues including colour and texture. For an illuminated corrugated, textured surface the shading pattern comprises modulations of luminance (first order, LM) and local luminance amplitude (second-order, AM). The phase relationship between these two signals enables layer decomposition, predicts the perception of reflectance and illumination changes, and has been modelled based on early, fast, feed-forward visual processing (Schofield et al., 2010). However, while inexperienced viewers appreciate this scission at long presentation times, they cannot do so for short presentation durations (250 ms). This might suggest the action of slower, higher-level mechanisms. Here we consider how training attenuates this delay, and whether the resultant learning occurs at a perceptual level. We trained observers to discriminate the components of plaid stimuli that mixed in-phase and anti-phase LM/AM signals over a period of 5 days. After training, the strength of the AM signal needed to differentiate the plaid components fell dramatically, indicating learning. We tested for transfer of learning using stimuli with different spatial frequencies, in-plane orientations, and acutely angled plaids. We report that learning transfers only partially when the stimuli are changed, suggesting that benefits accrue from tuning specific mechanisms, rather than general interpretative processes. We suggest that the mechanisms which support layer decomposition using second-order cues are relatively early, and not inherently slow.

## Highlights

► Second-order cues normally support layer decomposition at long but not short presentation times. ► Perceptual learning is used to train observers to perform the task at short presentation times. ► The transfer of learning is consistent with low level changes in perceptual processing.

## 1 Introduction

Interpreting the luminance variations in an image in terms of their underlying physical cause poses a significant challenge to the visual system. Specifically, luminance variations in an image can have two distinct causes: (i) they might arise from variations in 3D surface geometry so that different portions of a surface are differentially illuminated by the light source(s) and/or (ii) they might arise from variations in the surface albedo, such as different textures or paint on the surface. Somehow, the visual system should parse changes caused by the illumination with respect to the 3D surface (shape-from-shading) from changes in surface reflectance properties. This process is known as layer decomposition (Kingdom, 2008) or intrinsic image extraction (Barrow & Tanenbaum, 1978) and it can be achieved by considering the relationship between luminance variations and a range of other cues including colour (Kingdom, 2003) and, as we review below, second-order cues that arise in objects with a textured surface.

**Fig. 1:**
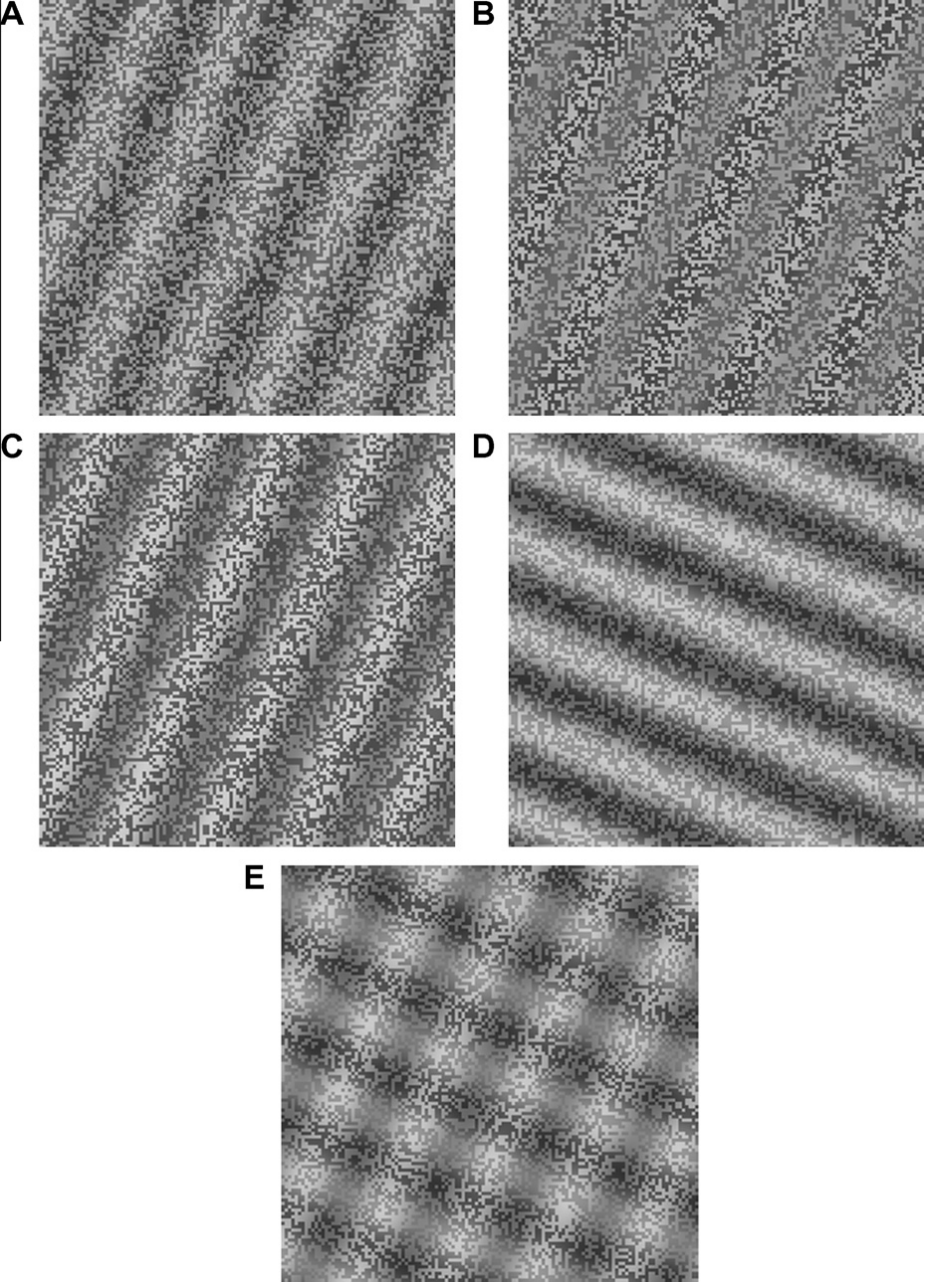
Stimulus examples (A) LM-only: A 45 deg oriented sine wave luminance grating added to binary noise (B) AM-only: Amplitude modulated binary noise pattern (modulation depth = 0.40). (C) An in-phase composite grating where peaks of LM (highest luminance) and AM (highest amplitude) are superimposed. (D) An anti-phase grating where LM troughs are superimposed with AM peaks. (E) A plaid consisting of an in-phase grating on the right diagonal (LM + AM) and an anti-phase grating on the left diagonal (LM − AM).

A potentially informative cue to layer decomposition is provided by the spatial relationship between changes in local mean luminance (LM) and local variations in the range of luminance values that arise from an albedo texture: local luminance amplitude (AM; Schofield et al., 2006). In particular, when the illumination varies across an albedo textured surface, changes in local mean luminance (LM) are positively correlated with changes in local luminance amplitude (AM). Adding an albedo texture to a shaded surface, such that LM and AM correlate positively (in-phase; LM + AM), enhances the impression of depth (Schofield et al., 2006, 2010; Todd & Mingolla, 1983; compare Fig. 1A and C). Further, if LM and AM are negatively correlated (anti-phase; LM − AM) the impression of depth is reduced (compare Fig 1D with A). If both relationships are present in a plaid configuration, the in-phase paring appears as a shaded undulating surface whereas the anti-phase paring appears as a flat material change (Schofield et al., 2006, 2010; Fig. 1E). The enhanced shape-from-shading in the in-phase case may be due to improved layer decomposition due to the information provided by the relative phase of the AM cue.

The changes in local luminance amplitude described above are, mathematically, closely related to the contrast modulations typically used to study second-order vision. The human visual system is known to be sensitive to second order signals and it is thought that they are detected separately from first order cues (Baker, 1999; Dakin & Mareschal, 2000; Ellemberg, Allen, & Hess, 2006; Fleet & Langley, 1994; Schofield & Georgeson, 1999, 2003). First- and second-order information are correlated in natural images (Johnson & Baker, 2004) but the sign of this correlation varies (Schofield, 2000), suggesting that contrast/amplitude modulations are informative by virtue of their relationship with luminance variations.

Building on the physiological work of Zhou and Baker (1996), Schofield et al. (2010) developed the shading channel model in order to explain the role of AM in layer decomposition. In this model LM and AM are initially detected separately and then recombined in an orientation/frequency specific additive sum that broadly mimics Zhou and Baker’s (1996) envelope neurons. In-phase parings sum to produce an enhanced output (greater perceived depth); whereas anti-phase parings subtract, weakening the output/depth percept. However AM components are given a relatively low weighting at the summation stage such that their effect on single LM components is marginal. A competitive gain control mechanism working across orientations produces the dramatic scission found for plaid stimuli. The model has been used to describe a range of psychophysical results (Schofield et al., 2010; Sun & Schofield, 2011), has been applied directly to natural images (Schofield et al., 2010), and has been used as the basis for a machine vision system for layer decomposition (Jiang, Schofield, & Wyatt, 2010).

The shading channel model relies on relatively low-level mechanisms, which might be considered comparable to envelope neurons found in area 17/18 of cat visual cortex (Zhou & Baker, 1996). Therefore we would expect layer decomposition based on LM and AM mixtures to be automatic and fast acting. Indeed, there are many examples of fast processing of textured stimuli for similarly complex tasks such as: estimating shape from texture (Gurnsey et al., 2006); detecting and discriminating second-order texture contrast (Barbot, Landy, & Carrasco, 2012); enhanced detection of orientation modulations when first- and second-order cues are combined (Johnson et al., 2007); and rapid scene identification (Renninger & Malik, 2004). However, whereas LM and AM combinations support layer decomposition for naïve participants at relatively long presentations times (ca. 1s), anecdotally they see no difference between LM + AM and LM–AM at short presentation times (250 ms): even in the more robust plaid condition. We confirmed this failing in Experiment 1. Thus layer decomposition is rather slow, implying the use of attentional mechanisms or at least multiple stages of processing beyond those implied by the shading channel model. However, it is also possible that early mechanisms exist for layer decomposition based on LM/AM mixtures but that they are either (i) relatively underused or (ii) not well engaged by plaid stimuli that are too different from everyday experience to allow fast layer decomposition. If this were the case we would expect performance to improve with training. Further if low level mechanisms, such as those described in the shading channel model, are critical to the task we would expect any benefits of learning to follow the stimulus specific pattern observed in perceptual learning studies (e.g. Fahle & Morgan, 1996; Jeter et al., 2010, 2009; Sagi, 2011).

Perceptual learning has been explored in various visual contexts. For instance, through repetitive training humans improve in their ability to: detect luminance contrast (Fiorentini & Berardi, 1981; Sowden, Rose, & Davies, 2002), perform Vernier tasks (Fahle & Morgan, 1996; Spang et al., 2010), discriminate between orientated stimuli (Jeter et al., 2009), and discriminate between textures (Karni & Sagi, 1991). Such perceptual learning is often reported to be specific to ancillary stimulus features such as retinal location, spatial frequency or orientation. For example Fiorentini and Berardi (1980) reported rapid learning in a phase discrimination task where observers are asked to discriminate the two types of composite sinusoidal gratings. This learning effect was specific for the trained orientation and did not transfer across 90 deg stimulus rotations. We might expect to see similar cue specific learing in the case of the relative phase discrimination required in our layer decomposition task.

We used a perceptual learning paradigm to examine the improvement in layer decomposition associated with LM/AM plaids at short presentation times. We then tested for transfer across stimulus dimensions as a marker for perceptual learning. We hypothesised that training would enable layer decomposition at short presentation times. Moreover, we test the generalisation of learning that results from training – considering the stimulus dimensions of orientation and spatial frequency. In particular, we trained naïve observers to discriminate the phase relationship of LM and AM signals in briefly-presented plaid stimuli. We then conducted three tests to probe the learning, examining the transfer of depth discriminations based on LM + AM to different stimulus rotations (Experiment 1), different stimulus spatial frequencies (Experiment 2), and plaids that differed in the relative orientation of their compositions (Experiment 3). Poor transfer across these stimulus manipulations would indicate perceptual learning whereas full transfer would suggest the learning of cognitive strategy such as labelling based on LM/AM relationship regardless of the percept formed by the stimuli.

## 2 Method

### 2.1 Stimuli

To isolate shading and illumination cues from additional sources of shape information (e.g. boundaries, occlusions or recognisable object outlines), we imposed sinusoidal modulations of luminance (LM, Fig. 1A) and amplitude (AM, Fig. 1B) on binary noise textures (see Schofield et al. (2006) for full details of the stimulus preparation method). Luminance and amplitude modulations could be aligned either in-phase (LM + AM, Fig. 1C) or out-of-phase (LM–AM, Fig. 1D) and could be oriented at four different angles (22.5, 67.5, 112.5 and 157.5 deg) with respect to vertical. Plaids were formed from combinations of an in-phase and an anti-phase grating presented at different orientations (Fig. 1E, in-phase on the right diagonal and anti-phase on the left diagonal). The orientations of the plaid components were orthogonal except in Experiment 3.

The contrast of all LM components was set to 0.2 in all experiments. AM values in the main experiment were chosen from an interval that would bound individual AM detection thresholds based on previous work (Schofield & Georgeson, 1999) and a pilot study: AM = 0.040 to 0.244 in five logarithmic steps. The spatial frequency of the modulations was 0.5 c/deg, except in Experiment 2. The noise contrast was fixed at 0.1 and new noise samples were generated for each trial. Stimuli subtended 6.5° by 6.5° of visual angle (256 × 256 pixels).

There were two training sets. Half the participants were trained on Set 1 (22.5 deg and 112.5 deg plaids) and the other half on Set 2 (67.5 deg and 157.5 deg plaids); the allocation of participants to training sets was random. We describe the plaids with respect to the orientation of the LM + AM component; thus, the 22.5 deg plaid contained the LM + AM component on the left diagonal, oriented 22.5 deg, and an LM − AM component on the right diagonal, oriented 112.5 deg. Having two training sets allowed us to examine the transfer of learning to untrained but otherwise similar stimuli. Component orientations were chosen to allow us to reasonably ask observers to judge whether the left or right tilted component was more corrugated.

Stimuli were generated in the frame store of a VSG graphics card (CRS Ltd, UK) with custom software written in C++ and were presented on a ViewSonic P225f monitor at a refresh rate of 160 Hz. The monitor’s gamma nonlinearity was estimated using a ColourCal luminance meter (CRS Ltd., UK) and corrected using the VSG’s lookup tables.

### 2.2 Participants

A total of 12 postgraduate students from the University of Birmingham took part in the study. Six participants (mean age = 29 ± 6 years) were tested to assess baseline performance using the test stimuli of Experiments 1 and 3 without any training. Another set of six participants (mean age = 25 ± 3 years) undertook the plaid training followed by the three test experiments. One of the participants showed a reverse learning effect during the first training session. She gave the opposite responses to those that were reinforced by the feedback; this observer was excluded from further study and replaced by a new participant given that we set out to study perceptual learning and this participant was unable to benefit from the feedback we provided. Participant GM was excluded from analysis in Experiment 3, as we could not estimate thresholds for him in 2 out of the 3 conditions. All of the participants were naïve to the purposes of the experiment, and had normal or corrected to normal vision for our viewing distance/good accommodation. Participants gave written informed consent and were paid £6 per hour. They were debriefed after the last test session. The work was subject to ethical review by the University of Birmingham ethics committee prior to experimentation.

### 2.3 Procedure

The stimulus duration was 250 ms for each condition. Stimuli could appear in one of two locations either 1.5 deg above or below the fixation marker; the other location was filled with a binary noise pattern. This manipulation prevented the build up of afterimages, which can selectively reduce the visibility of the LM cue. Participants viewed the stimuli in a darkened room at a viewing distance of 0.6 m. Head position was stabilized with a chin rest. Participants indicated whether the right or the left oblique seemed more corrugated in depth by pressing one of the two keys on a button box (CB3, CRS Ltd., UK). Given previous results (Kingdom, 2003; Schofield et al., 2006, 2010), responses for ‘In-phase component has greater depth’ were counted as hits. Symbolic, intermittent feedback was given: specifically, at the end of each block of 20 trials, observers were shown their per cent correct score for the last block via a written message on the display. The first 200 trials on Day 1 of training were analysed separately to represent the performance levels for initial exposure to the stimuli. Training continued for 5 days (1000 trials per day). In cases where overall accuracy was below 75% correct after 5 days (participants GM, JB, and AM), training continued until an overall accuracy of 75% correct was achieved (longest duration: 10 days). Trained participants undertook three experiments to test their newly learnt abilities in the days immediately following training.

### 2.4 Post-training experiments

After training, participants made forced-choice judgements (“*Which orientation in the plaid is more corrugated in depth?*”) on 3 sets of test stimuli: (1) stimuli were orthogonal plaids differing from the training stimuli by a 45 deg rigid rotation (Fig. 1E) participants who were trained on Set 1 stimuli were presented with Set 2 stimuli to establish performance on untrained stimuli, and vice versa; (2) spatial frequency (Fig. 4A, s.f. = 2 or 4 c/deg, angle between components 90 deg); or (3) shear (Fig. 5A, angle between the LM + AM and LM − AM components varied while still allowing left, right judgements to be made). No feedback was given during the test phase. Experiment 1 took place 1 day after the final day of training; Experiment 2, 9–13 days post training; and Experiment 3, 15–19 days post training.

## 3 Results

### 3.1 Performance during training

**Fig. 2:**
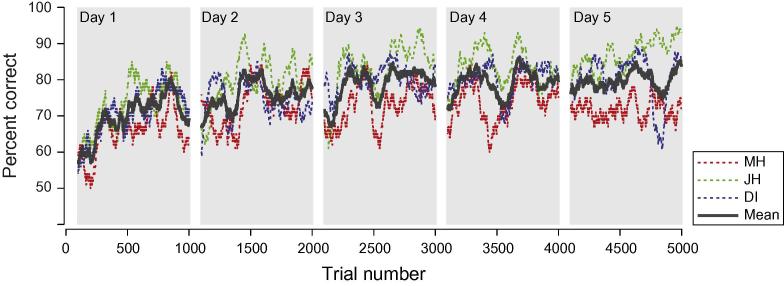
The time course of training. Lines show trial-by-trial percent correct scores calculated over the preceding 100 trials starting from the 100th trial in each session. Accuracy was assessed relative to ‘in-phase has greater depth’ this being deemed the correct response. Gray boxes show each day’s training. Green, blue and red traces show results for participants MH, DI and JH respectively. The black line shows the mean performance of the three participants. (For interpretation of the references to colour in this figure legend, the reader is referred to the web version of this article.)

As a first analysis, we considered the efficacy of the training paradigm on participants’ behavioural performance. In particular, we considered trial-by-trial performance during training for the three observers who completed the training regime within 5 days (Fig. 2). We calculated the proportion correct (later converted to percent correct) as a running average, based on a window of the preceding 100 trials (1 = correct = ‘in-phase component has most depth’, 0 = incorrect) for each day of training. (Performance on each day is described from the 100th trial, therefore there are gaps in the traces between each day). On the first training day, performance improved up to a peak at around 80% correct and but fell dramatically in the last 200 trials perhaps due to fatigue or reduced participant confidence due to a run of weak stimuli. Such dips occur elsewhere in the data and are not confined to the last trails of a session. Performance at the start of day two was above that at the outset of day one but below the day one peak. Performance on subsequent days showed progressively increasing initial performance with smaller lapses from the previous day. By day five, initial performance was consentient with the overall mean. The remaining observers showed similar training performance but were slower to reach the asymptotic performance and showed bigger initial drops in performance.

### 3.2 Experiment 1: Specificity for orientation

Following the training phase, we examined whether improvements in depth judgments were specific to the trained stimuli. Experiment 1 tested for transfer between different stimulus orientations. In particular, we tested for the transfer of performance between the trained and untrained stimulus sets, which differed in overall orientation by 45 deg.

**Fig. 3:**
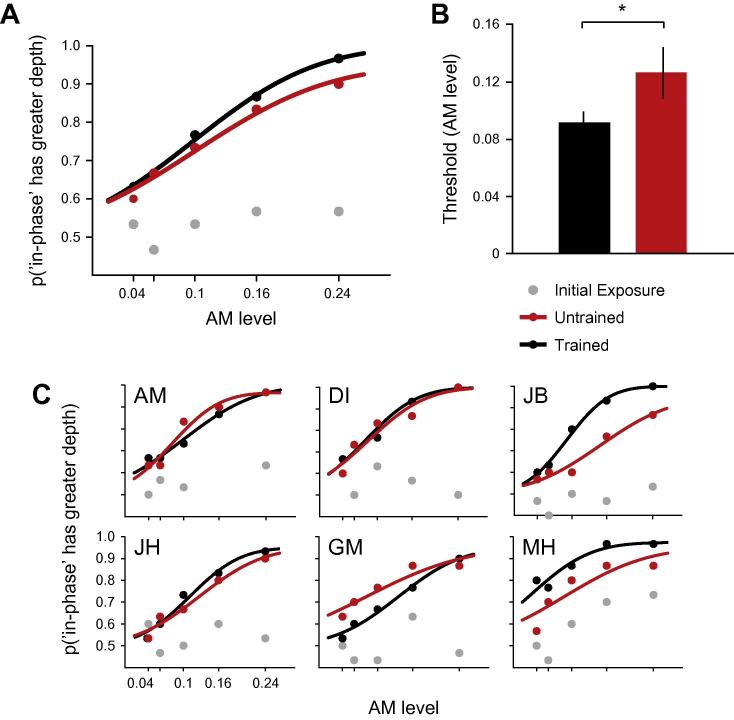
Plaid training and Experiment 1 – specificity for trained orientation: (A) Mean psychometric functions (6 participants), grey data points indicate per cent correct for initial exposure to plaids, black symbols (line) data (fit) for trained stimuli, red symbols (line) data (fit) for untrained stimuli. (B) Thresholds (75% correct) for AM level required to discriminate phase relationship for trained (black bar) and untrained (red bar) orientations. (C) Individual psychometric functions, each graph represents data from a single participant on three conditions as described in (A). (For interpretation of the references to colour in this figure legend, the reader is referred to the web version of this article.)

Fig. 3A shows the percent correct values and a fitted cumulative Gaussian function (mean of six new observers; fits obtained using psignifit version 2.5.6; Wichmann & Hill, 2001) for the trained and untrained stimulus sets. Performance during initial exposure to stimuli (grey data points, see also Supplementary Fig. 1) is around chance, suggesting that untrained observers cannot differentiate LM–AM from LM + AM at short presentation durations. This was also confirmed for 6 new, untrained observers (Supplementary Fig. 2). However, after training (black and red lines) observers were able to determine that the LM + AM component had a greater corrugation than the LM − AM component, with average performance reaching up to 96 ± 4% correct at the highest AM level for trained stimulus orientation (black dots). The difference between thresholds for trained and untrained stimuli shows that the learning effect is somewhat specific to the trained stimulus set (Fig. 3B). In particular, thresholds for the trained stimuli (black bar, AM = 0.09 ± 0.01) were significantly lower than the untrained stimulus thresholds (red bar, AM = 0.13 ± 0.02; *t*(5) = 2.11, *p* = .044, *d* = .86). However, post-training thresholds for untrained stimuli were better than pre-training thresholds (which were not measureable) suggesting a partial transfer of training. Indeed two participants (GM and AM) perform slightly better on the untrained stimuli suggesting complete transfer for these individuals. To provide an index of training transfer that could be compared across experiments, we converted thresholds to sensitivity and then divided sensitivity to untrained stimuli by that for trained stimuli (Table 1). Using this index, we found that there was 73% transfer of training across rigid translations of the stimuli.

### 3.3 Experiment 2: Specificity for spatial frequency

**Fig. 4:**
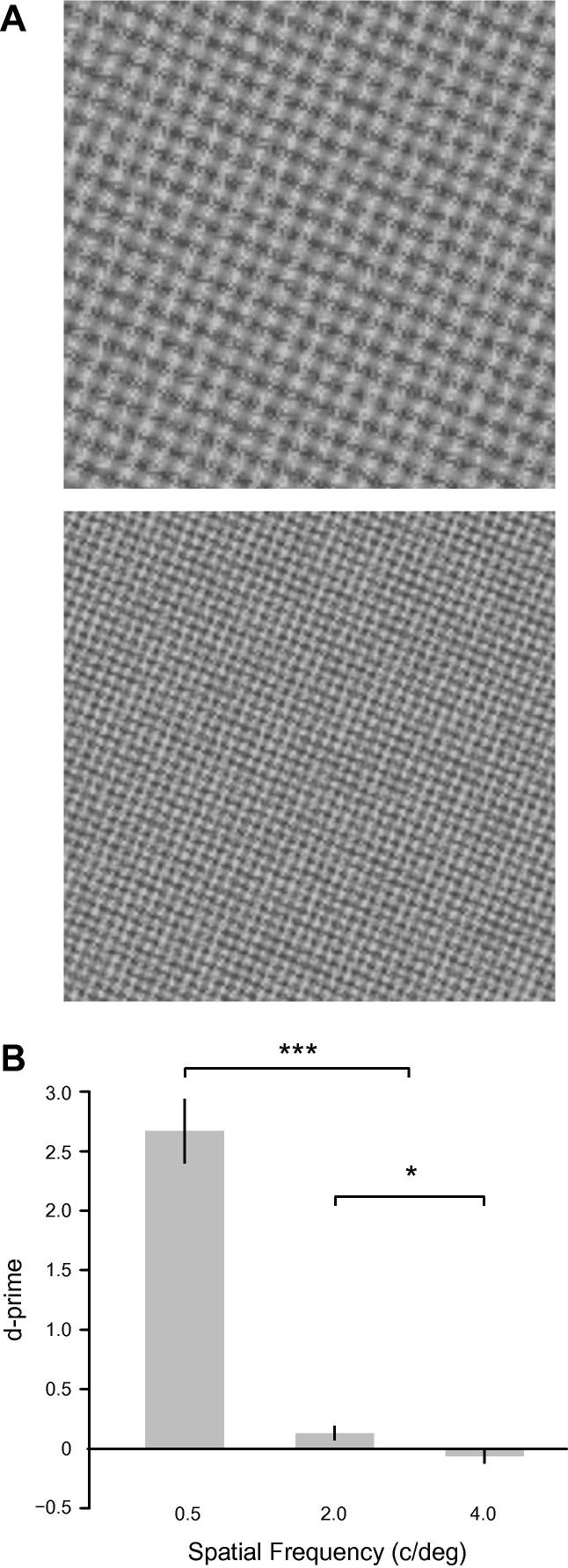
Experiment 2 – no transfer across spatial frequency: (A) Example stimuli for 2 c/deg (upper) and 4 c/deg (lower) plaid. Both plaids are oriented at 22.5 deg, i.e. in-phase component is on the right diagonal. (B): Performance for high spatial frequency plaids (2 c/deg and 4 c/deg) at the highest AM level compared to the equivalent performance for 0.5 c/deg for the trained stimuli in Experiment 1. Error bars indicate ±S.E.M. and asterisks indicate where the difference is significant (*** for *p* < .001 and * for *p* < .05).

In Experiment 1 the full benefits of training were specific to the trained orientation, with only partial transfer to 45 deg rigid rotations. Here we test transfer along another stimulus dimension: spatial frequency. Detection thresholds for LM and AM cues depend on spatial frequency in different ways (Schofield & Georgeson, 1999) but relative and absolute sensitivity for the two cues is approximately equal at 0.5 and 2 c/deg in the presence of binary noise. That is LM (or AM) thresholds are similar at the two frequencies and the ratio of LM to AM sensitivity is also similar at the two frequencies. In this experiment, we tested whether training at 0.5 c/deg transfers to 2 c/deg plaids (Fig. 4A, top image). We also tested for transfer to a higher spatial frequency (4 c/deg; Fig. 4A, bottom image) where AM sensitivity is known to be relatively weak. Only the strongest AM level (0.244) was used in this experiment. Other stimulus dimensions were the same as the training stimuli and the task was the same as in the general methods.

Percent correct values were converted to *d*′ to indicate the discrimination sensitivity for LM/AM phase relationship (in- or anti-phase) at the highest AM level (0.244). Fig. 4B shows the mean performance across six observers. Data from Experiment 1 (0.5 c/deg, trained orientation, AM = 0.244) is shown for comparison. Repeated measures ANOVA showed that there was no transfer of training with 0.5 c/deg stimuli to 2 or 4 c/deg stimuli (main effect spatial frequency, Greenhouse–Geisser corrected, *F*_1.0,5.0_ = 100.7, *p* < .001, $\eta_{p}^{2}$ = .95). Bonferroni corrected comparisons also showed differences in mean *d*′ values for 2 c/deg vs. 4 c/deg (*p* < .05). The transfer index for this experiment was calculated as the ratio of *d*′ values for trained and untrained stimuli (see Table 1). The percent correct measure for 4 c/deg was less than 50% giving a negative *d*′. We can safely assume that this represents sampling error on a true *d*′ of zero. Hence we have recorded the transfer index for 4 c/deg as zero. There is no evidence of transfer across spatial frequency.

### 3.4 Experiment 3: Partial transfer to non-orthogonal plaids

**Fig. 5:**
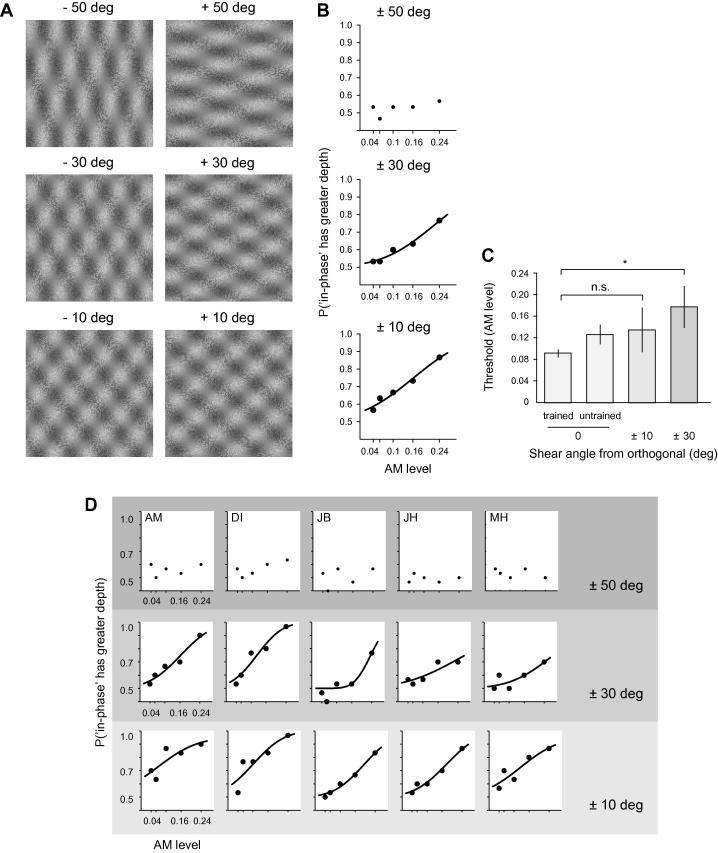
Experiment 3 – Partial transfer to non-orthogonal plaids: (A) Stimulus examples used in Experiment 3. Different shear angles are shown (rows) where left and right columns separate negative and positive shears, respectively. (B) Mean psychometric functions for six participants. Each plot shows percent correct data points and psychometric functions (where available) as shear angle decreases from top to bottom. (C) Mean thresholds for AM level at 75% correct (‘in-phase has a greater depth’) rate from six participants are presented for ±30 deg shear (darker grey bar) and ±10 deg shear (lighter grey bar); it was not possible to extract threshold for ±50 deg shears. Data from Experiment 1 (white bars, 0 deg shears) are added for comparison. Error bars indicate ±S.E.M. and asterisk indicates a significant difference (*p* < .05). (D) Individual plots show psychometric functions for each participant (columns) on three groups of shear angles (rows). Shear angles decrease from top row to bottom row, hence becoming more similar to the training stimulus (shear = 0 deg).

Experiment 1 showed that training for a single orientation of plaids did not fully transfer to 45 deg rigid rotations of orthogonal plaids. Here we investigate whether the training effect is specific to the angle between the components of the plaids in training sets. The plaids used in the training were all orthogonal; here we rotated the two components in a plaid separately so that their combination was no longer orthogonal. We introduce *shear angle* to define non-orthogonal combinations of the in- and anti-phase components. That is, if a plaid has a shear angle of +10 deg then the angle between the two components is 100 deg; whereas orthogonal plaids have 90 deg between their components and hence a shear angle of 0 deg. In Experiment 3, participants viewed non-orthogonal plaids with 6 shear angles (−50, −30, −10, 10, 30, 50 deg; Fig. 5A) at all five levels of AM. All other stimulus parameters were as described in the general methods.

It was not possible to fit psychometric functions for shear angles +50 or −50 deg, because participants performed around chance even for the highest AM signal in these conditions (Fig. 5B, top graph). A repeated measures ANOVA with 2 factors (shear sign and magnitude) showed a significant main effect of magnitude (*F*_2,8_ = 31.93, *p* < .0001, $\eta_{p}^{2}$ = .89) but no effect of sign (*F*_1,4_ < 1, *p* = .42, $\eta_{p}^{2}$ = .17) and no interaction (*F*_1.0_,_4.0_ < 1, *p* = .729, $\eta_{p}^{2}$ = .04), therefore we grouped shear angles according to their magnitudes: ±10, ±30, ±50. Fig. 5B shows average percent correct (see supplementary Fig. 3 for *d*′) values at each AM level and fitted cumulative Gaussian psychometric functions (where possible) for three different groups of shear angle magnitudes of non-orthogonal plaids.

Thresholds decreased for smaller absolute shear angles but they were still higher than the thresholds obtained for trained stimuli in Experiment 1 (mean ± st. dev. for ±10 deg: AM = 0.14 ± 0.04 and for ±30 deg: AM = 0.18 ± 0.04). Fig. 5C shows mean thresholds alongside data from Experiment 1, where one can see that thresholds increase as the shear increases. These results show that smallest shear angles did not differ from trained plaids when we compare thresholds (trained plaid vs. ±10 deg shear: *t*(4) = 1.81, *p* = .078, *d* = .81), so the benefit of training transferred to ±10 deg shears. Experiment 3 was conducted last in the test sequence so this result also shows that the lack of transfer in experiment 2 cannot be due to a return to the untrained state over time. Thresholds for ±30 deg are significantly higher than plaid thresholds for the trained stimulus set in Experiment 1 (*t*(4) = 4.36, *p* = .006, *d *= 1.95). However, statistical analysis showed that the thresholds for ±10 and ±30 deg were not significantly different (*t*(4) ⩽ 1, *p* = .373, *d* = 15). Table 1 shows transfer indices for Experiment 3 calculated as the ratio of sensitivities. Thresholds were un-measureable for 50 deg shears so we have not calculated a transfer index for this condition. Partial transfer of between 50% and 70% was observed for small shear angles with no transfer at larger shears. Supplementary Fig. 4 shows *d*′ values for untrained observers in the sheer task showing again that the task is impossible without training.

## 4 Discussion

The luminance variations in a scene are potentially ambiguous. They could be caused by changes in the light source position, changes in illumination due to surface orientation or shadows, or they can represent intrinsic properties of the viewed surface such as albedo reflectance. For example, a surface might have different colours or it may consist of different materials so that its reflectance changes. Schofield et al. (2006, 2010) have shown that the phase relationship between first-order luminance modulations (LM) and second-order amplitude modulations (AM) can be used to discriminate luminance dependent changes from reflectance dependent changes: in-phase combinations give rise to the percept of a corrugated surface via shape-from-shading while anti-phase combinations appear as reflectance changes. Schofield et al. (2010) proposed the shading channel model as a mechanism by which AM can influence the perceived role of luminance variations in an image. This model relies on early visual mechanisms and suggests that layer decomposition using these cues should be automatic and quick. However whereas naive participants can use the relationship between LM and AM at relatively long presentations times (Schofield et al., 2006, 2010) they fail to do so at shorter presentation times.

Here we have shown that layer decomposition based on the phase relationships of LM and AM cues in plaid stimuli can be achieved at short presentation times (250 ms) following training with intermittent feedback. This decomposition was specific to the trained stimulus and did not fully transfer to plaids at other orientations (Experiment 1). It transferred for small shear angles of non-orthogonal plaids (Experiment 3). However, training did not transfer at all to higher spatial frequency plaids (Experiment 2) or to larger shear angles for non-orthogonal plaids, even though the AM cue was as visible in such test stimuli as it was in the trained stimuli.

In the initial exposure phase of plaid training, we showed that observers are not able to differentiate LM + AM from LM − AM at brief presentation times. After 5–10 days of training with intermittent feedback, performance improves and observers start judging LM + AM as more corrugated than LM − AM. This suggests that observers learn to make use of the AM cue and its alignment with LM (in- or anti-phase) as cues to shape from shading; they learn to segment shading dependent illumination changes from material dependent changes. In other words, they learn to see the difference caused by the alignment of the AM cue; judging the anti-phase aligned LM/AM combination as a flat surface.

In Experiment 1, we also used novel stimuli to test whether the benefit of training transfers across rigid rotations. Performance on novel plaids was better than that at initial exposure; however thresholds remained significantly higher than those for trained plaids. The transfer index of 73% suggests partial transfer of training across rigid rotations.

The results of Experiment 2 indicate that observers could not make use of the AM cue or phase relationship to differentiate shading from reflectance changes in high frequency stimuli. The ability to use the AM signal failed to transfer to 2 c/deg or 4 c/deg plaids. AM sensitivity varies with spatial frequency; as does that for LM, but, based on the sensitivity functions found by Schofield and Georgeson (1999), we would not expect any marked change in the visibility of either cue between 0.5 and 2 c/deg when binary noise is present. So the change in performance cannot be due to a lack of visibility for the AM cue at 2 c/deg and must rather reflect an inability to combine the cues or make use of the relative phase information.

The spatial configuration of LM + AM and LM − AM components in a plaid seems to be important for layer decomposition. Specifically the LM − AM component in a plaid with an orthogonal LM + AM component is seen as a very flat reflectance change whereas it is seen as moderately corrugated when presented alone. We now show that the orthogonal configuration is itself important. Even when trained in the layer decomposition task participants cannot discriminate the two components when the plaid is sheared by 50 deg thus reducing the minimum angle between the two components to 40 deg. Transfer across smaller shear angles is only partial. These results indicate that training is specific to the alignment of the two components in the plaid.

Overall, our findings provide evidence for stimulus specific perceptual learning of the layer decomposition task based on the phase relationship of LM/AM mixtures at short presentation times. This supports the shading channel model proposed by Schofield et al. (2010). The ability to perform the tasks described in this paper at short presentation times strongly suggests that the task is supported by early, automatic, mechanisms. The failure of transfer across stimuli properties (specifically spatial frequency) confirms that learning took place at a perceptual rather than cognitive level again implicating low level mechanisms.

We should, however, consider why fast layer decomposition is available only after training. According to the shading channel model, cross-orientation gain control is fundamental to the perceptual scission between LM + AM and LM − AM cues in the plaid condition. One possibility is that this mechanism, which most likely relies on feedback loops, is normally quite sluggish but that its action can be speeded with training via a strengthening of the inhibitory links. Gain control mechanisms are known to be relatively broadband so this reasoning may explain the partial transfer that we found in some conditions. It should also be noted that the gain control mechanisms seems to be less useful in natural stimuli than in our plaid stimuli (Schofield et al., 2010) and that the machine vision system proposed by Jiang, Schofield, and Wyatt (2010) dispenses with it altogether. Thus the human visual system might not normally deploy the cross-orientation gain control mechanism implied by the shading channel model but might engage it when repeatedly presented with the plaid decomposition task.

In summary, we have shown that layer decomposition based on the phase relationship of LM and AM cues can be achieved at short presentation times only after training and that this training is characterised a perceptual rather than cognitive learning. These findings support an account of layer decomposition based on early, automatic processes although training may be required to tune these processes to deal with specific experimental stimuli.

## Funding support

Supported by the EPSRC (EP/F026269/1 to AJS) and a Wellcome Trust fellowship (095183/Z/10/Z to AEW). The funders played no direct role in the study.

## Figures and Tables

**Table 1:** Transfer index. For experiments 1 and 3 the transfer index was calculated as the ratio of sensitivity (1/threshold) to untrained and trained stimuli. For experiment 2 the *d*′ ratio was used.

Experiment 1 Rigid rotations	Experiment 2 Spatial frequency		Experiment 3 Shear	
	2 c/deg	4 c/deg	±10°	±30°
.73	.05	0	.68	.52

## Appendix A Supplementary material

Supplementary data 1 Supplementary material contains ‘Figs. 1–4’.
