# Supplementary material for: Perceptual learning of second order cues for layer decomposition
Source: Vision Res. 2013 Jan 25;77:1–9. doi: 10.1016/j.visres.2012.11.005 (PMC3552157; doi:10.1016/j.visres.2012.11.005)
Supplement: Supplementary data 1 — Supplementary material contains ‘Figs. 1–4’. [file mmc1.doc]

# Perceptual learning of second order cues to Shape-from-Shading

**Dicle N. Dövencioğlu, Andrew E. Welchman & Andrew J. Schofield**

**Supplementary figures**


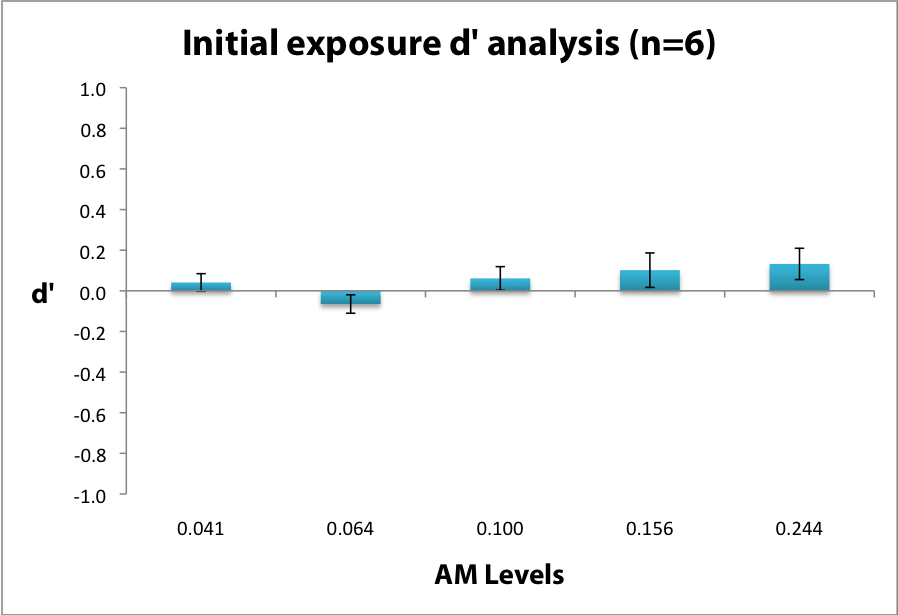


Figure 1: Averaged d' values from first 200 trials of the training phase. These results are shown as percent correct data points in Experiment 1 results. Error bars indicate ±1 SEM.


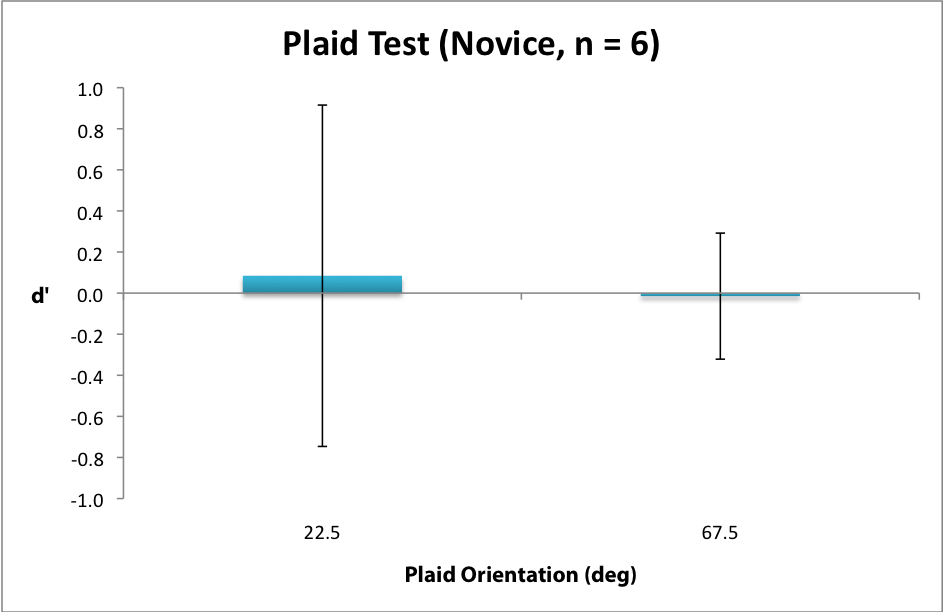


Figure 2: Replication of Experiment 1 with untrained observers d’ values averaged across all participants (AM = 0.24). Error bars indicate ±1 SEM.


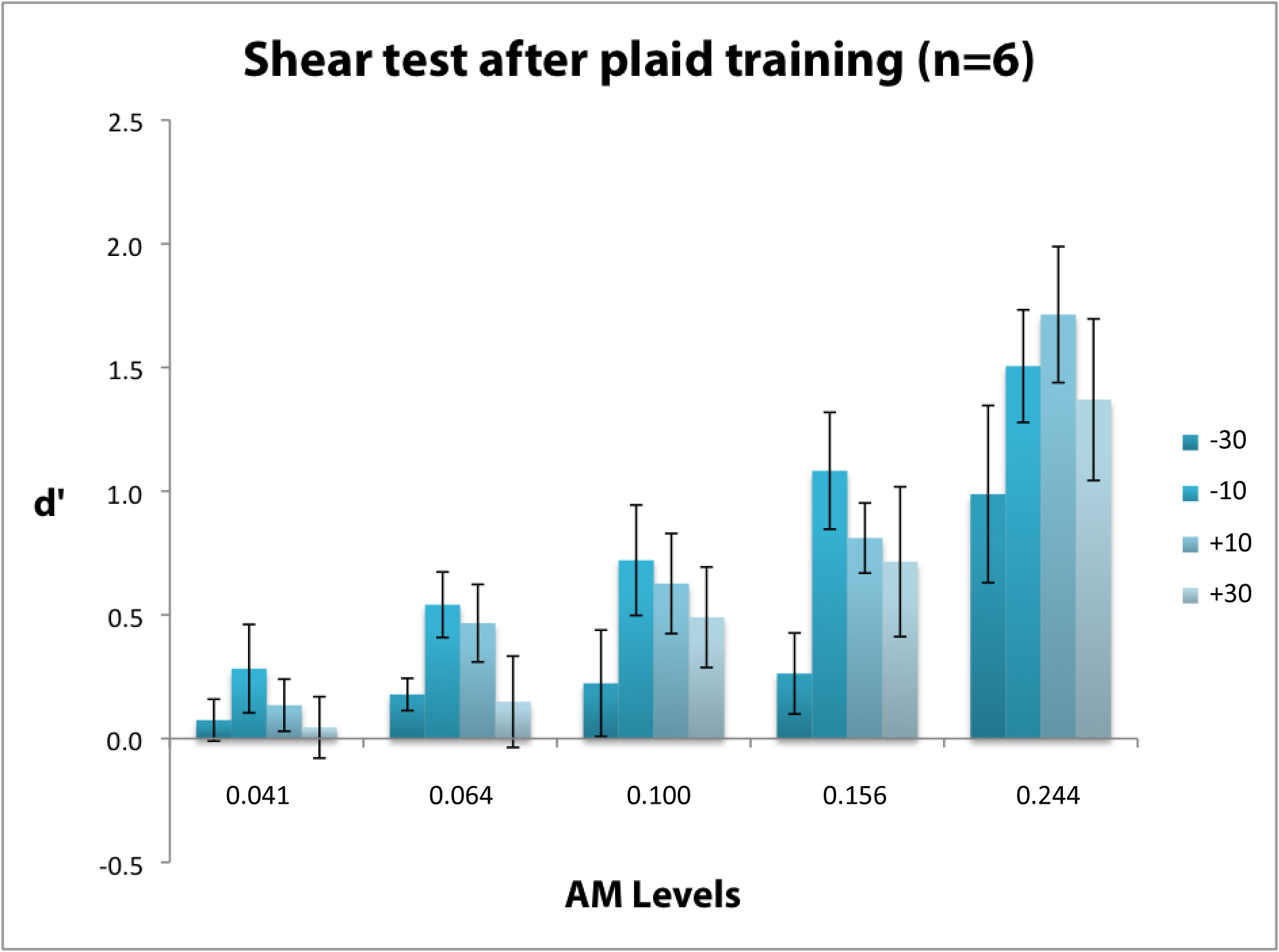
Figure 3: Experiment 3 - Averaged d' values for each stimulus shear type and AM signal strength. In the manuscript, these results are presented as fitted Cumulative Gaussian functions to average percent correct data points on each AM level. Error bars indicate ±1 SEM.


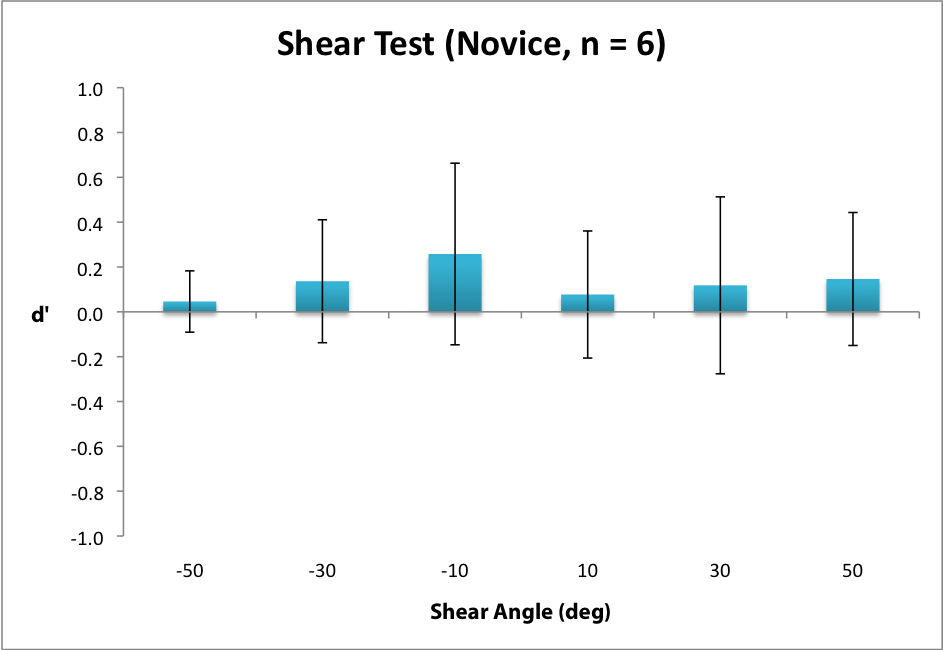


Figure 1: Replication of Experiment 3 with untrained observers - Sensitivity for AM = 0.24 on 6 different non-orthogonal plaids. Error bars indicate ±1 SEM.
